# Facing the Challenge of Data Transfer from Animal Models to Humans: the Case of Persistent Organohalogens

**DOI:** 10.1186/1476-069X-7-58

**Published:** 2008-11-13

**Authors:** Alexander Suvorov, Larissa Takser

**Affiliations:** 1 Département Obstétrique Gynécologie, Faculté de Médecine et des sciences de la santé, Université de Sherbrooke, 3001, 12 avenue Nord, Sherbrooke, Québec, Canada, J1H 5N4

## Abstract

A well-documented fact for a group of persistent, bioaccumulating organohalogens contaminants, namely polychlorinated biphenyls (PCBs), is that appropriate regulation was delayed, on average, up to 50 years. Some of the delay may be attributed to the fact that the science of toxicology was in its infancy when PCBs were introduced in 1920's. Nevertheless, even following the development of modern toxicology this story repeats itself 45 years later with polybrominated diphenyl ethers (PBDEs) another compound of concern for public health. The question is why? One possible explanation may be the low coherence between experimental studies of toxic effects in animal models and human studies. To explore this further, we reviewed a total of 807 PubMed abstracts and full texts reporting studies of toxic effects of PCB and PBDE in animal models. Our analysis documents that human epidemiological studies of PBDE stand to gain little from animal studies due to the following: 1) the significant delay between the commercialisation of a substance and studies with animal models; 2) experimental exposure levels in animals are several orders of magnitude higher than exposures in the general human population; 3) the limited set of evidence-based endocrine endpoints; 4) the traditional testing sequence (adult animals – neonates – foetuses) postpones investigation of the critical developmental stages; 5) limited number of animal species with human-like toxicokinetics, physiology of development and pregnancy; 6) lack of suitable experimental outcomes for the purpose of epidemiological studies. Our comparison of published PCB and PBDE studies underscore an important shortcoming: history has, unfortunately, repeated itself. Broadening the crosstalk between the various branches of toxicology should therefore accelerate accumulation of data to enable timely and appropriate regulatory action.

## Background

The history of research on a group of persistent, bioaccumulating organohalogen environmental contaminants, namely polychlorinated biphenyls (PCBs), shows serious delays in the accumulation of necessary data for the purpose of toxic-substance policy making and regulatory action [1]. Delays averaging some 50 years, depending on the country, have brought on far-reaching consequences with damage to human health, natural ecosystems and the economy [2]. The ever-increasing accumulation of industrial contaminants in biota, the influence of which is of yet unknown to human health, is one reason why speeding up policy making and regulatory action is becoming an important goal in the field of toxicology.

Could delays in regulatory action be at least partly attributable to the low coherence between experimental studies of toxic effects in animal models and human studies? In other words, to what extent the outputs of animal studies aid in the design of human studies? Animal models play an important role in studying the toxic effects of hazardous substances, determining dose-response relationships, and pinpointing the most susceptible developmental stages. The results of animal experiments form an important input for human studies which influence directly the policy making process. Any discordance between them may slow down the regulatory process. Hence, an important question is to what extent the outputs of existing animal experiments correspond to the input requirements for human studies?

To answer this question, we compared the animal-study histories of two groups of well known organohalogen contaminants: PCBs and PBDEs (polybrominated diphenyl ethers). PCBs have been relatively well studied compared to PBDE which are a relatively new group with a much shorter history of studies. In addition, we also ask what lessons, if any, are to be drawn from history?

We reviewed PubMed titles, abstracts and full texts selected with the terms "PCB" or "PBDE" as search criteria on May 15th, 2008. All reports that dealt with animal model experiments were selected then by reading available texts (titles/abstracts/full texts). The following reports of experiments with animal models were excluded from the subsequent analysis: (1) *in vitro *experiments with animal cells and tissues; (2) reviews; (3) experiments whose exposure was not controlled (field studies, exposure by use of contaminated food with unknown concentration of organohalogens, etc); (4) strictly toxicokinetic reports not addressing health effects; (5) experiments using various metabolites and derivatives of PBDEs and/or PCBs; (6) experiments using heterogeneous mixtures of contaminants (i.e. mixtures consisting not only of PCB or PBDE congeners). Mixtures of PBDEs or PCBs congeners were included. In other words, only reports of *in vivo *animal experiments addressing health effects of controlled exposure to PCBs or PBDEs were selected. No other restrictions were applied. 748 out of a total of 6076 PCB articles and 59 out of 649 PBDE articles met our selection criteria and were retained for analysis (see Additional files 1 and 2). All the data used further in this study were extracted from titles, abstracts or full texts of all selected articles with the priority given to more complete text.

It should be noted that PubMed databases do not effectively capture the older scientific literature that predated the internet and existed only in print media. Thus, the first PCB studies we analysed were from 1971 [3,4] although PCBs were commercialised in the 1920's. This limitation is not so essential for analysis of PBDE papers due to their much later arrival in the market. To avoid bias resulting from limitations of electronic databases, the data are expressed as a percentage of total articles in a given time period.

Our study is not a systematic review but rather reflection based on analysis of history. We analyse existing experiments with animals in order to see if the level of coherence with human studies can be a limitation for the development of the latter.

### What factors are important to permit the transfer of animal data to human studies?

In an attempt to approach existing animal data in a systematic fashion and determine whether they are compatible with existing and ongoing epidemiologic investigations on environmental contaminants, we designed a non-exhaustive set of basic requirements in order to better "harmonize" animal experiments for the purpose of data transfer to human studies.

When considering animal toxicology studies with a view to timely toxic-substance policy making and regulatory action, "harmonized" experiments with animal models should fulfill at least six basic requirements. These are summarized below.

1. Experiments must be completed long before the substance can become a public health concern and well enough in advance to allow timely policy making and regulatory action. Obviously, once a persistent, toxic substance has already accumulated in the environment, as was the case with PCBs, the problem is greatly compounded.

To study time-trends in animal research we analysed the temporal distribution of all selected 748 PCB and 59 PBDE reports using the print publishing date with respect to the dates of the beginning of production, initial detection in human tissues, and subsequent ban.

2. The doses studied must be relevant to human exposure levels. Toxic effects and biomarkers drawn from exposures several fold higher than the levels of exposure of the general human population cannot be transferred directly to the studies of the general population since most of toxic substances exert different effects at different doses. Moreover, toxic effects obtained from dose-response studies performed at high doses cannot be extrapolated to existing low-dose exposures of the general population. This is so because a number of environmental toxicants display non-linear dose-response relationships. Non-monotonic dose-response curves were already reported for PCBs [5-7] and for PBDEs [8-10]. Baseline corticosterone concentrations in American kestrels (Falco sparverius) exposed to commercial mixture of PCBs (Aroclors) exhibited a hormetic response in relation to total PCB liver burden [5]. In one assay [6] rainbow trout exposed to low dose (0.4 μg/egg) of another commercial PCB mixture (Clophen A50) through eggs nanoinjections showed decreased disease (trout fry syndrome) resistance while resistance of group exposed to high dose (2 μg/egg) was not significantly different from the control group. Perturbations in the hypothalamic-pituitary-thyroid axis were highly nonlinear with respect to PCB 126 dosing in study by Fisher *et al *[7]. According to Gee and Moser [10] number of open-field rears during a 1-minute observation period was significantly higher in male mice exposed neonatally to a single oral dose of 1 mg/kg BDE-47 than in control group as well as mice exposed to 10 and 30 mg/kg BDE-47. Talsness et al [8] reported the increase in ovarian weight in rats received prenatally 140 μg/kg bw but not 700 μg/kg of BDE-47. In our recent study [9] pups exposed perinatally to 0.002 mg/kg BDE-47 appear to be much more hyperactive on PND 20 than groups exposed to 0.2 and 0.02 mg/kg. Biphasic and hormetic dose-response curves are widely discussed in the toxicology literature [11,12] suggesting, for a majority of cases, the involvement of heterogeneous mechanisms of response to different threshold concentrations. Experimental testing exclusively at only high doses can therefore lead to a false sense of security with respect to the safety of a substance for the general population.

In short, the use of doses several fold higher than exposure levels for the general human population contributes little to our understanding of the risks incurred by that population.

To analyse doses of PCB and/or PBDE used in animal experiments we extracted, when available, data on dosing protocols from all selected PCB and PBDE reports, namely dose of compound per individual administration (daily dose) and total number of administrations. Total dose was calculated by multiplying daily dose by number of administrations. An average daily and average total dose was calculated for each year as mean ± SE for all experiments reported in the respective year. In number of cases, more than one experiment was presented in single paper. We estimated as separate experiments, trials with animals within one paper if they differed at least in one of the following: animal species/strains, treated stages of development, administered compounds, daily exposure doses and total exposure doses.

3. The most sensitive endpoints must be screened, i.e. health effects emerging at the lowest exposure levels. Adequate risk assessment for the general population must be based on the analysis of toxic effects on the most sensitive target organs and functions. Obviously it is impossible to start research of new substance with unknown properties with the most sensitive endpoints. It is thus necessary to screen a series of endpoints to identify the most sensitive as well as to use previously studied substances with similar structures (PCB for PBDE) to predict possible endpoints.

To detect health effects in the general population exposed to low doses of environmental contaminants, biomarkers of effect are essential. Biomarker epidemiology is undergoing a rapid development and expansion and is quickly becoming one of the most promising areas of environmental research [13]. Animal studies enable broader possibilities in the search of toxicity endpoints than does epidemiological research on human populations. Indeed it allows for the sacrifice of animals at any stage of development, tissue harvesting as well as various other types of interventions. Also animal experiments are not as costly and robust as epidemiological studies. Thus, animal studies must uncover non-invasive biomarkers of low dose exposure suitable for human studies.

Therefore, comprehensive screening of the most sensitive endpoints and peripheral biomarkers of exposure is another necessary requirement for animal studies.

We extracted data on the health effects of PCB or PBDE exposure for each experiment of each selected article when available and assigned them according to a slightly modified classification of the health effects used in ATSDR's (US Agency for Toxic Substances and Disease Registry) Toxicological Profiles. All effects were classified among the following groups: mortality, respiratory, cardiovascular, gastrointestinal, haematological, musculoskeletal, hepatic, renal, endocrine, dermal, ocular, metabolic, other systemic, immunological and lymphoreticular, neurological, reproductive, developmental effects, cancer and genotoxicity. We modified the classifications with respect to two aspects. First, endpoints dealing with endocrine activity of reproductive organs were considered as endocrine rather than reproductive effects. Second, all endpoints addressing different organs at early stages of development were assigned to the effect corresponding to the organ rather than development and only growth changes were considered as developmental, including developmental malformations, developmental landmarks and endpoints addressing foetal development.

4. The most sensitive stages of development must be screened. A number of substances (including well-known examples of ethanol and thalidomide) are known developmental toxicants at low doses but do not display toxic properties for adults at the same concentrations. Therefore, the most sensitive stages of development must be screened so as avoid underestimation of the risks posed to the most susceptible and vulnerable populations. Obviously, it is impossible to start research on a new substance with unknown properties with the most sensitive stages. It is necessary however to screen of most sensitive ages as well as use already studied substances with similar structure (PCB for PBDE) to predict them. The particular sensitivity of early developmental stages to PCB exposure was shown in number of animal and human studies (see reviews [14-17]). PBDE was shown recently to be of particular concern for early stages of development as being potent neurodevelopmental toxin [18] and because of higher levels of exposure in children than in adults [19-22].

We extracted data on animal stages of development exposed to PCB or PBDE from all selected articles when available and assigned them to one of the following: prenatal exposure; neonatal exposure; perinatal exposure; adult exposure. All these types of exposures were further united into two groups: developmental exposures (pre-, neo- and perinatal) and adult exposures. The temporal distribution of experiments with different types of exposure was analysed.

5. Ideally animal models should have human-like physiology and toxicokinetics. Differences in toxicokinetics predetermine different toxic effects in different species [23]. For example, Geyer *et al *[24] estimated that BDE-47 has a terminal half life of 664 days in humans using daily intake and total-body burden data. Terminal half life for the same PBDE congener in mouse was calculated to be 23 days and it is rapidly excreted from the organism due to mechanisms of active transport [25]. This difference in toxicokinetics complicates transfer of health effects data obtained in experiments with mouse to human studies, while experiments with mouse average one third of total number of published animal studies.

It is becoming increasingly clear that prenatal and neonatal stages of development are highly susceptible to a number of toxic substances [1]. Therefore, not only is it important to use animal models with human-like adult physiology and toxicokinetics but also human-like pregnancy, pre-neonatal and neonatal development.

We extracted data on animal species used in each experiment reported in selected articles to analyse time-trend distribution of taxa in PCB and PBDE experiments.

6. Exposures must be verified by measurement of the internal dose. For animal data to transfer correctly to human studies, it is especially important to measure the internal dose since exposure levels are rarely measured in epidemiological studies and all the health effects are correlated with the internal dose.

There is no universally accepted protocol for the correct estimation of internal dose of exposure. In fact, there is a broad range of protocols using different tissues, time-points, dose-parameters and chemical species. PBPK models are of use also. Given the large number of protocols for determining of internal dose, we simply verified presence or absence of any estimation of internal dose in the selected articles.

We extracted from all selected papers, when available, data on estimation of internal dose of the administered substance in exposed animals. We considered that the internal dose had indeed been estimated if there was any type of measurement of residuals or metabolites of administered congeners done at any time point in any tissues of directly exposed animals or their offspring, in the case of developmental exposures.

We used these basic requirements in order to "harmonize" reports of animal experiments for the purpose of data transfer to human studies. Our short list is by no means exclusive and may be extended by others. We assume that applying such requirements to the analysis of animal data is the first step to document the level of coherence between animal and human studies.

We also added the route of exposure to our analysis of PCB and PBDE studies because it is, according to our personal experience, the subject of intense debate (in peer review processes) with experts often displaying widely varying opinions.

It is commonly accepted that studying the mechanisms of toxicity greatly increases the relative value of animal model studies. Albeit highly interesting in itself, knowledge of toxicity mechanisms impacts only slightly on policy making and regulatory action. For instance, in spite of some 30 years of intensive research, we are still unclear on the specific mechanisms of PCB toxicity. What is clear, however, is that this knowledge gap should not have precluded the ban on PCB in the 1970's. That is why our short list does not include analysis of toxicity mechanisms for harmonizing purposes.

### Short history of PCB and PBDE

Both PCB and PBDE are ubiquitous, persistent organohalogens. Until their ban in the 1970's, PCB accumulated in biota. The bioaccumulation of PBDE, which are still massively produced, continues.

PCBs was first manufactured commercially in 1927. They were used as coolants and insulating fluids for transformers and capacitors, as stabilizing additives in flexible PVC coatings of electrical wiring and electronic components, as pesticide extenders, as cutting oils, flame retardants, hydraulic fluids, sealants (used in caulking, etc), adhesives, wood floor finishes, paints, de-dusting agents, and in carbonless copy paper. Manufacture peaked in the 1960's when the total global production of PCB was estimated at 1.5 million tons per year [26]. In 1966, the Swedish chemist Dr. Soren Jensen designated PCB as ubiquitous environmental contaminants [27]. Global PCB atmospheric emissions peaked at an estimated 400 tons per year by the early 1970's [28]. General Electric (GE) alone had dumped between 0.5 to 1.5 million pounds of PCB into the Hudson River by 1976 [29].

PCB-associated toxicity was recognized quite early due to a variety of industrial incidents [30]. The most commonly observed health effects on people exposed to PCB are skin conditions such as chloracne and rashes. Studies on PCB-exposed workers report changes in blood and urine, possibly pointing to liver damage. In 1968, PCB contamination of rice bran oil in Japan caused mass poisoning and Yushô Disease in over 14,000 people [31]. Common symptoms included dermal and ocular lesions, irregular menstrual cycles, lowered immune response, fatigue, headaches, cough, and unusual skin sores. Poor cognitive development in children was reported at much lower doses [32,33].

PCB production was banned in most countries during the 1970's, some 50 years after its commercial introduction in the 1920's.

PBDE are a group of flame retardant chemicals which are added to synthetic polymers. PBDE are thus found in various building materials, electronics, furnishings, motor vehicles, plastics, polyurethane foams, and textiles. The first patent for PBDE use as a flame retardant was issued in 1960 and manufacturing of commercial products containing PBDE begins in 1965 [34] several years before the PCB ban. Total world demand for PBDE in 2001 was estimated at 67,440 metric tons by the bromine industry [35].

PBDEs were first detected in the environment in 1979 [36] and in biota in the 1980's [37]. Studies on North American wildlife report sharp increases in PBDE levels over a 20-year period, with levels doubling every 3 to 5 years [38-41]. In human blood, milk, and tissues, total PBDE levels have increased exponentially over the past 30 years, doubling every 5 years [42].

Although their health effects, especially at low doses, need to be studied further, PBDEs are nevertheless known to be associated with neurodevelopmental toxicity and thyroid hormone imbalance in rodents [18,43-47].

The European Union banned the use of penta- and octa-BDE in 2004 and recently banned deca-BDE in 2008. Penta-and octa-BDE were withdrawn from the North American market in 2004 [48] but Deca-BDE still remains in use in North America. Little is known with respect to PBDE production in Asian countries. Waste and recycling sites, indoor use of PBDE-containing products, and global circulation of PBDE toward the northern hemisphere from countries without policy, all contribute to the long term persistence of PBDE in Europe and North America.

The introduction of PBDE coincided with the development of the modern environmental movement inspired partly by the scandalous story of PCB and including the development of toxicological sciences, the establishment of public environmental institutions and environmental legislation in countries around the world.

However, more than 40 years elapsed between the start of PBDE production in 1965 and the ban of most of the PBDE congeners. Deca-BDE is still in use in North America, but unfortunately produces banned lower-brominated congeners by natural debromination *via *photochemical reactions [49-51], anaerobic processes in sludge and sediments [52,53] and metabolism in animals [54-56].

### Experimental timeline: too little too late?

**Figure 1:**
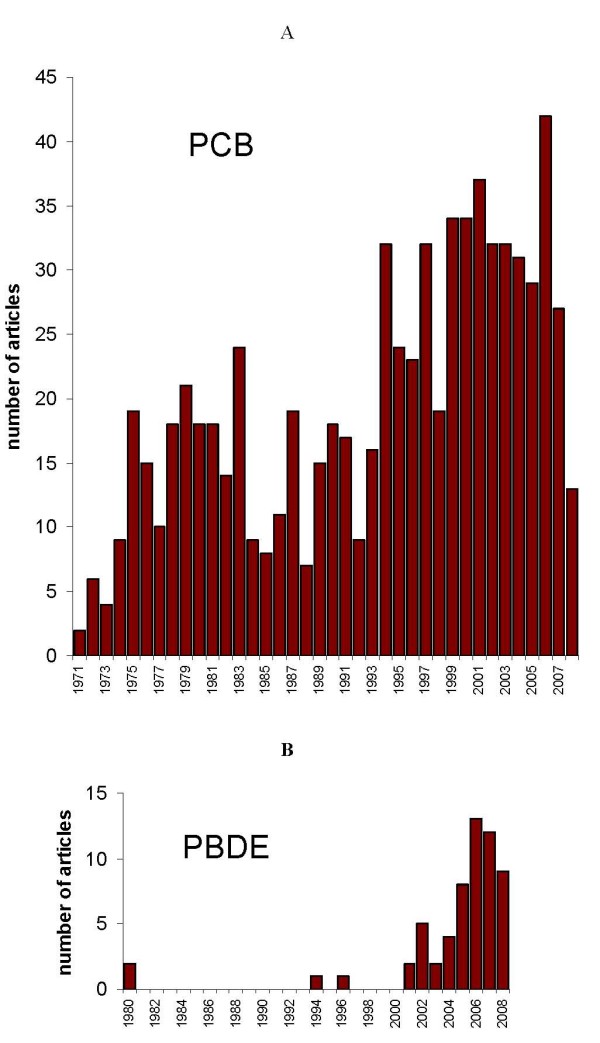
Temporal distribution of studies addressing PCB (A) and PBDE (B) toxicity in experiments with animal models.

The oldest experimental reports on PCB found through our PubMed search were published in 1971. This likely reflects the limitations of the electronic database rather than the first toxicological studies of PCB. The number of publications increased steadily every year thereafter (Fig 1A). The growing number of publications across the earlier years may reflect the increasing proportion of the published literature that is captured in PubMed as well as the actual trend of research changing over time.

It is noteworthy that the number of PCB reports continued to rise despite PCB ban (in 1976 in the USA; and 1970's in most PCB-producing countries). The number of publications peaked in 1984 and then again in 1994. The first wave of interest was presumably triggered by current exposures and observed toxic effects, whereas the next was consecutive to emerging results from long-term epidemiological studies on the general population.

The two oldest experimental studies on PBDE found through our PubMed search were published in 1980 [57,58], several years after the PCB ban. In spite of these reports of PBDE toxic effects and of the known structural similarity of PBDE and PCB, there were only two publications between 1980 and 2001 – one in 1994 [59] and another in 1996 [60] (Fig 1B). Renewed interest in PBDE has been sustained since 2001 (see also [34]).

Hence, intensive research only started once PBDE had become ubiquitous, with high concentrations in biota. In Sweden, PBDE concentrations in breast milk increased by approximately two order of magnitude during last 30 years [42] and relatively high concentrations of PBDE were found in almost every human and biota sample by the early 1990's.

To characterise actual rate of studies: 161 PCB papers and 44 PBDE papers (27% of number of PCB papers) were published during the last 5 years (2002–2007), while the ban was completed for PCB approximately 30 years before and was in the process for PBDE.

It took approximately 40 years to detect PCB in human tissues since the start of production. The discovery of the ubiquitous presence of PCBs in fat tissues of humans was an important step in the recognition of their potential danger. This discovery was delayed by the inability to measure PCB residues with sensitive analytical methods. The PCB designation as ubiquitous environmental contaminants occurred in 1966 [27], one year after the start of PBDE production. It explains the decrease with time until the discovery the ubiquitous presence of PBDE. The period from substance detection in human tissues until the ban accounted for approximately 10 years for PCB and more than 25 years for PBDE (banning is not yet completed for this substance). Hence, the development of sensitive analytical methods did not appear to have affected the pace of environmental regulation for these substances.

### Are study doses relevant to human exposure levels?

**Figure 2:**
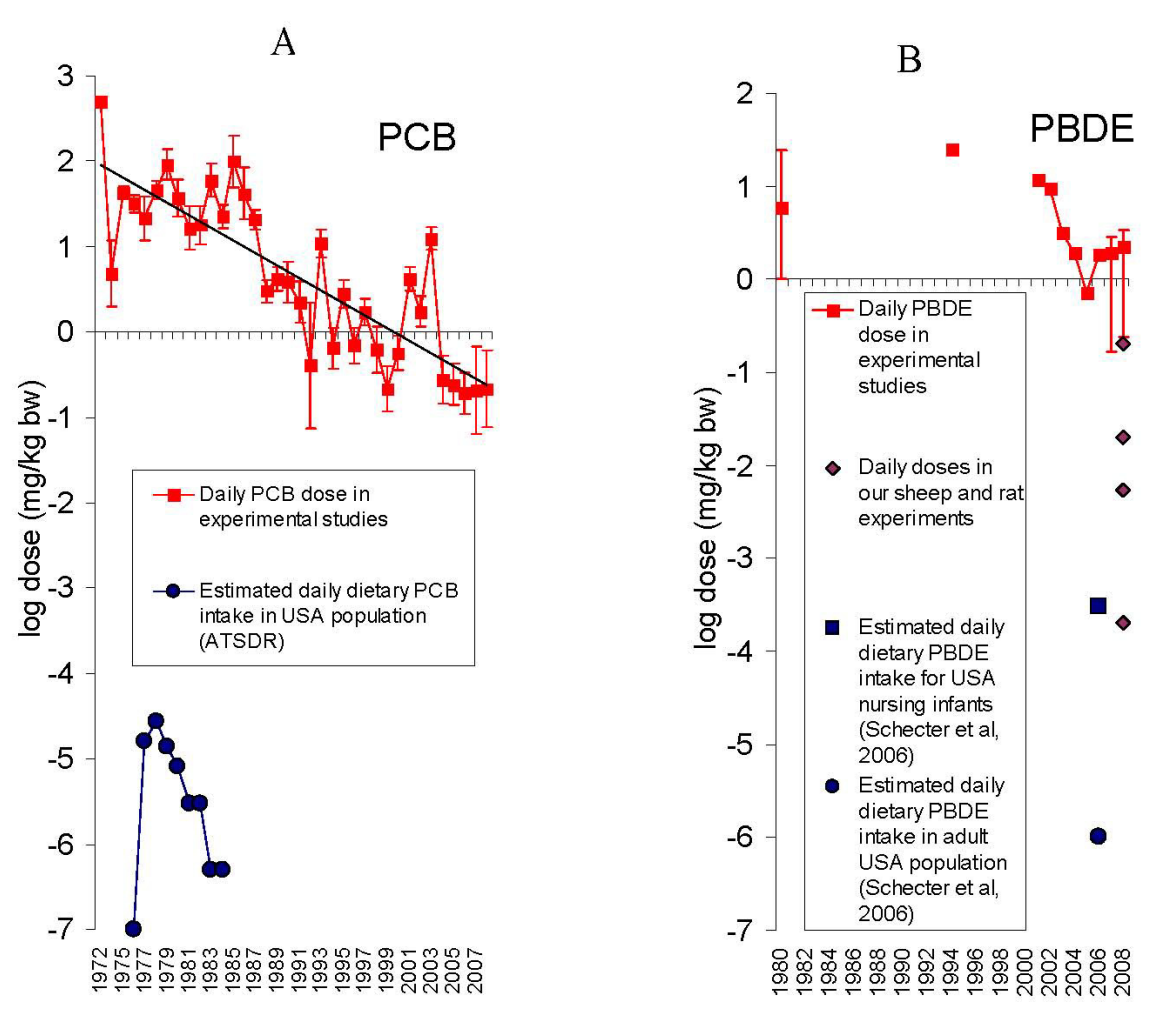
**Temporal trends toward decrease of average daily dose in experiments with animal models. ** A – experiments addressing PCB toxicity; B – experiments addressing PBDE toxicity.

Over a 30-year period, the average daily PCB doses used in experiments with animal models decreased steadily from hundreds of milligrams per kilogram of bodyweight (mg/kg bw) in the early 1970's, to hundreds of micrograms/kg bw presently (Fig 2A). Meanwhile, human dietary daily doses, at the period of highest exposure for the general population, were estimated by the ATSDR at tens of nanograms/kg bw. Daily exposure of the general population is now estimated at picograms/kg bw. Therefore, in spite of 30 years of decreasing PCB daily exposures, animal experiments are still performed with doses several orders of magnitude higher than human exposures. The resulting graph does not change when the average total dose is used instead of average daily dose (data not shown).

With the benefit of hindsight from the PCB experience, investigators would be expected to choose PBDE doses closer to human daily exposures. However, PBDE experiments were nevertheless initially conducted (Fig 2B) with high doses (tens of mg/kg bw) steadily decreasing to the hundreds of micrograms/kg bw in 2005. Doses began to rise once again despite the fact that the 2005 threshold was known to be far above human exposures levels. In the U.S., daily PBDE dietary intake in 2003–2004 was estimated by Schecter *et al*. [20] at 0.2 μg/kg bw for nursing infants, and 0.001 μg/kg bw for adults.

We hypothesize that doses increased after 2005 because the no observed adverse effect level (NOAEL) was reached at doses used in 2005. To test this hypothesis we tested the developmental effects of subchronic exposures at low doses of PBDE with rat and sheep animal models.

Pregnant rats were exposed to vehicle or low doses of BDE-47 (2, 20 and 200 μg/kg bw), the most prevalent PBDE congener found in human samples, by intravenous injections every 5 days, from gestational day 15 to postnatal day 20. Hyperactivity and decreased total thyroxine in offspring were observed in all tested groups [9]. Similarly, pregnant sheep were exposed to vehicle or BDE-47 (0.2, 2 and 20 μg/kg bw), by intravenous injections weekly, from the 5^th ^to the 15^th ^week of gestation. Decreased total triiodothyronine was observed in lambs of least exposed group upon delivery [61]. BDE-47 content in adipose tissue of mothers and offspring of both models was analyzed and found to be similar to that reported for human populations. The observed effects indicate that doses even several fold lower than those achieved in 2005 produce a number of adverse effects. In view of these results, one cannot help but wonder if it was the achieved NOAEL which incited investigators to increase levels of exposure in animal models, or rather the likelihood of "unpublishable" results?

### The quest for the most sensitive endpoints

Each new environmental contaminant poses the challenging quest for sensitive endpoints of toxicity. In spite of the large body of knowledge from toxicology, predicting the toxic properties of new contaminants on the basis of their chemical structure remains difficult. Systematic screening of the most sensitive endpoints is necessary for each potentially hazardous substance. The powerful new tools of "omics" research which appeared over the last decades will no doubt increasingly contribute to intensifying the investigation into the sensitive endpoints of toxicity. Two reports, both published in 2007, investigate PCB toxicity using microarray technology [62,63]. Another 2006 article reports the study of PBDE toxicity using proteomics [64]. All other articles reviewed tested different endpoints by traditional methods.

**Figure 3:**
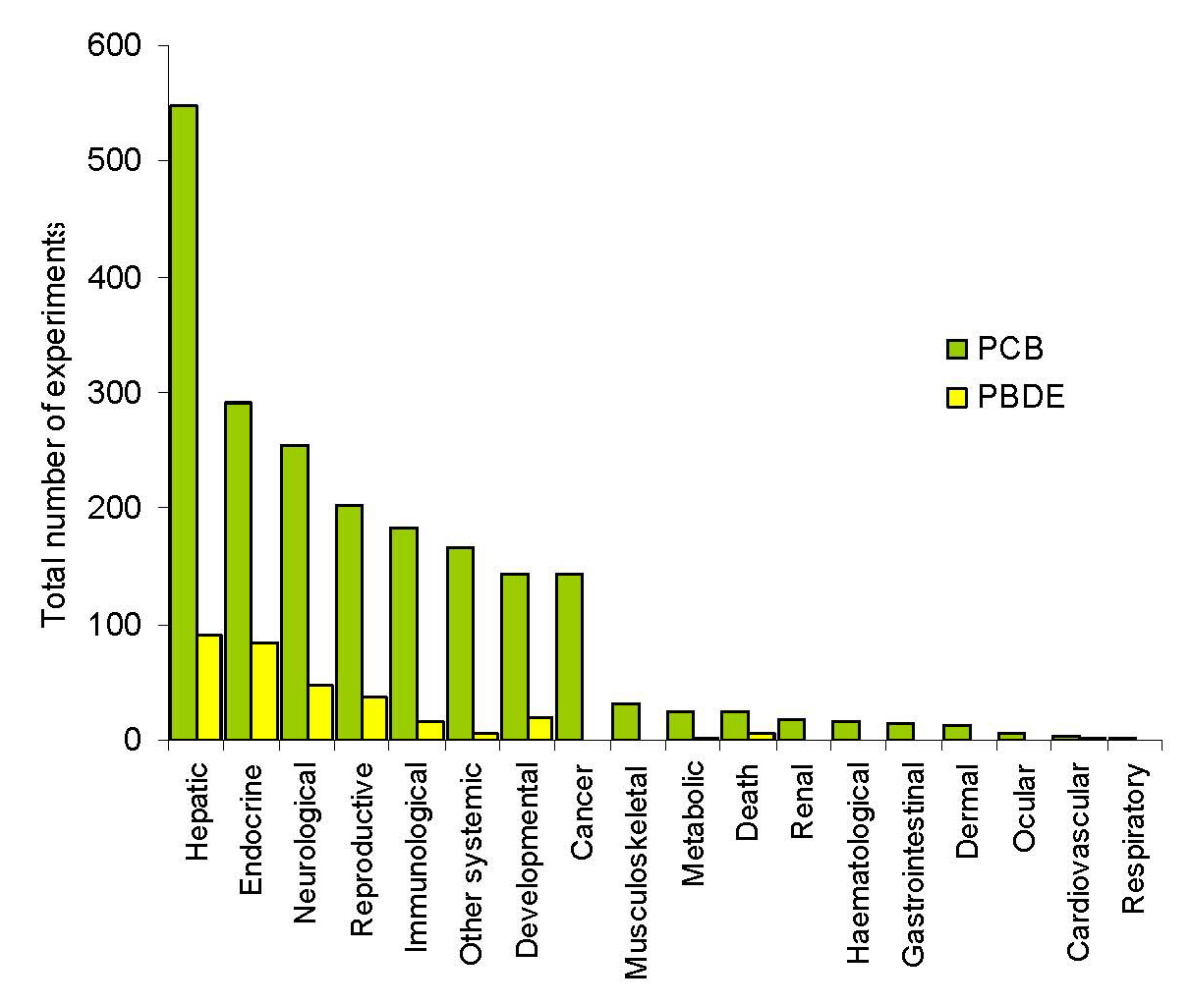
Distribution of endpoints of PCB and PBDE toxicity addressed in experiments with animal models.

In general, similar profiles of endpoints are addressed in PCB and PBDE studies (Fig 3). The range of endpoints is relatively broader for PCB studies while experiments addressing cancer, dermal, gastrointestinal, haematological, musculoskeletal, ocular, renal, and respiratory effects are missing in PBDE studies. One possible explanation for this could be the shorter history of PBDE studies and the smaller total number of experiments.

Surprisingly, in spite of the important number of experiments on PCB carcinogenicity, information on the carcinogenicity of PBDE in animal models is still lacking. Although a number of studies indicate that certain congeners exhibit aryl hydrocarbon receptor (Ah-R)-mediated effects and are potent inducers of ethoxyresorufin-o-deethylase (EROD), direct studies of the carcinogenic properties of PBDE have yet to be reported.

**Figure 4:**
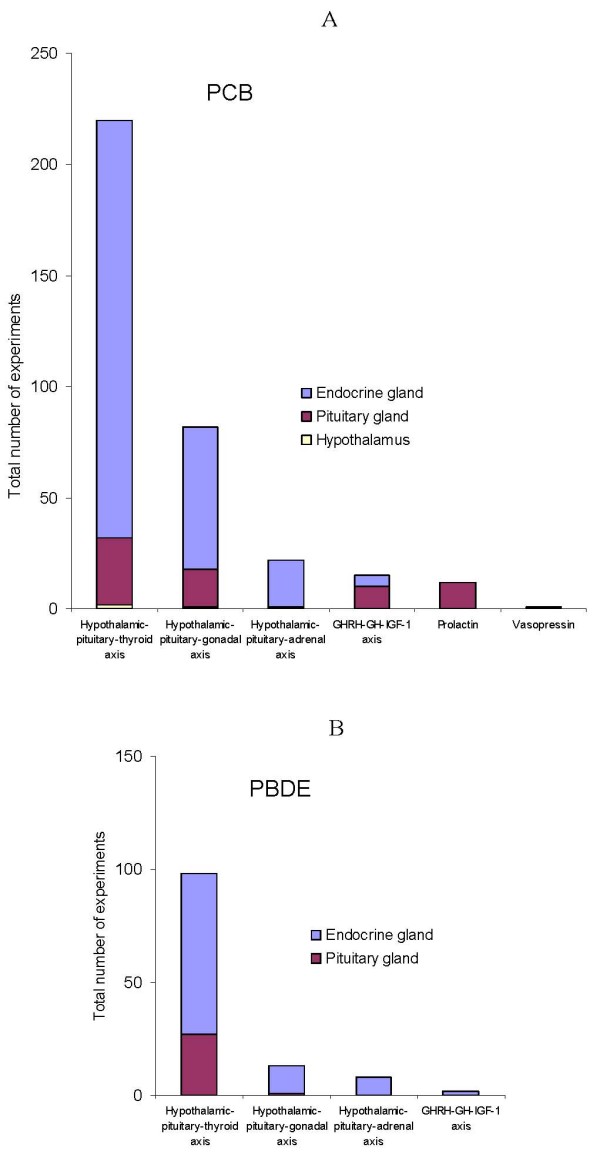
Distribution of endocrine endpoints of PCB (A) and PBDE (B) toxicity addressed in experiments with animal models.

Because of the well known endocrine disruptive properties of organohalogens, we considered endocrine endpoints in more detail (Fig 4). Despite the large number of reports on endocrine endpoints, the diversity itself of the studied endpoints is relatively limited and, surprisingly, the same in PCB and PBDE studies. A disproportionately large number of experiments address the hypothalamo-pituitary-thyroid axis (202 for PCB and 98 for PBDE studies), whereas only a few studies address the hypothalamo-pituitary-adrenal axis (22 for PCB and 8 for PBDE studies). This is surprising in view of the fact that the adrenal gland: a) secretes hormones which regulate most processes in the human organism, and b) is the target for environmental PCB [65]. Research into the effects of PBDE on the adrenal gland is thus seriously lacking. But why is this so?

One possibility is that the adrenal gland is insensitive to organohalogens. To test this hypothesis, we studied adrenal endpoints of low-dose PBDE toxicity in our developmental subchronic exposure rat model described hereinabove [9]. In the offspring of group exposed to 0.2 mg/kg bw BDE-47 we observed decreased corticosterone, adrenal atrophy, impaired adrenal zonation and impaired expression of steroidogenic enzymes. These results show that the adrenal gland is probably among the most sensitive targets of PBDE toxicity. Hence, lack of attention to the adrenal gland, as opposed to the thyroid gland, could be due simply to tradition.

Numerous PCB and PBDE studies report a correlation between level of exposure and different biomarkers of effect which, theoretically, could be used in human studies. However a closer analysis of these outcomes indicates three major limitations. First, the range of peripheral biomarkers of toxicity is limited. Second, the overwhelming majority of experiments use exposure levels several fold higher than exposure levels in the human population. Outcomes are thus effects of acute toxicity rather than effects of chronic low-dose exposure characteristic of the general population. Lastly, most outcomes determination requires sacrifice of animals and thus they are inapplicable to human studies, such as measurements of liver microsomal enzyme activities. Hence, our findings are in perfect concordance with ATSDR Toxicological Profile for PCB: "*There are no specific biomarkers of effects for PCBs. Numerous studies have attempted to correlate serum PCB levels with liver-associated enzymes in PCB-exposed workers and general population subjects; however, no conclusive association has been found *[66-77]. *Further studies to identify specific biomarkers of effects of PCBs would facilitate medical surveillance leading to early detection of potentially adverse health and possible treatment*." (p. 499, [78]). Only thyroid hormone is indicated in the profile for PBDE [79] as a biomarker of effect with the notation that it is highly non specific.

It has to be mentioned that all the references cited in the above paragraph are attributed to epidemiological studies. Our review of 807 PCB and PBDE experimental reports also revealed no single study especially designed and aimed at the investigation of outcomes suitable for epidemiological studies. This further underscores the lack of integration of the various branches of the field of toxicology.

### Screening for the most sensitive stages of development: a paradigm shift?

It was shown for a number of environmental contaminants, including PCB and PBDE, that the early stages of development are much more sensitive than adulthood [80,81]. Thus top-priority should be given to experiments aimed at screening for the most sensitive stages of development in order to identify and protect the most vulnerable segment of the human population. However, this represents an important shift for researchers and brings into question traditional testing sequences.

**Figure 5:**
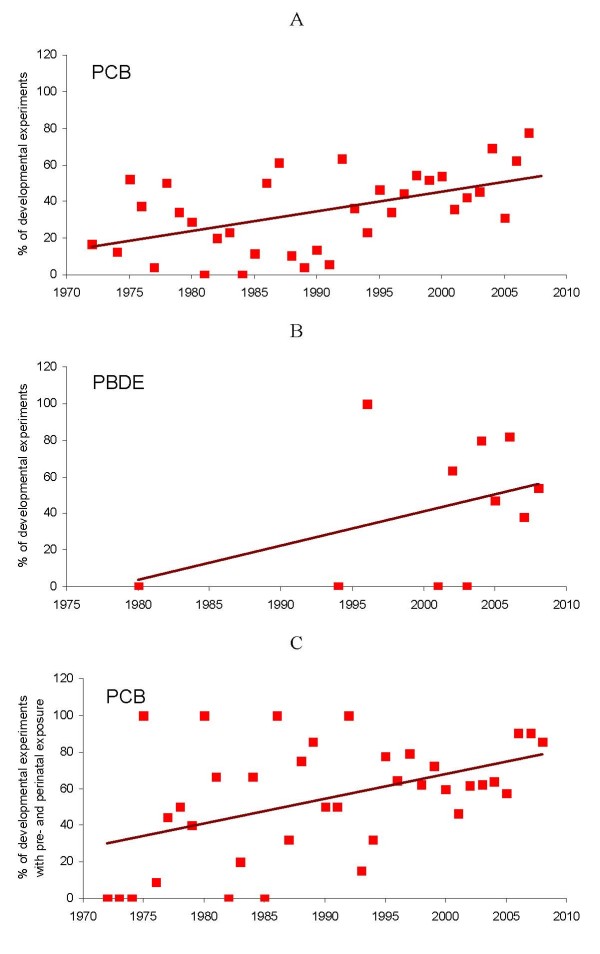
**Trends towards increase in number of developmental experiments *versus *experiments with adult animals.** A – developmental experiments addressing PCB toxicity; B – developmental experiments addressing PBDE toxicity; C – increase in number of pre- and perinatal versus neonatal exposures within developmental experiments with PCB.

The ratio of developmental *versus *non-developmental experiments per year are summarized in Figure 5. The proportion of studies using developmental models instead of adult testing increased gradually in PCB studies (*β *= 1.08; *p *= 0.01), the same trend was observed for PBDE reports (*β *= 1.87; *p *= 0.16). Moreover, the number of pre- and perinatal exposures *versus *neonatal exposures increased within PCB developmental experiments (*β *= 1.36; *p *= 0.004). The number of PBDE reports is too small to conclude any similar trend.

Hence, investigators test adult animals before the more susceptible neonates. Foetuses, which are in fact the most susceptible, are tested last whereas they should logically be tested first.

### Animal models: are we using enough species?

**Figure 6:**
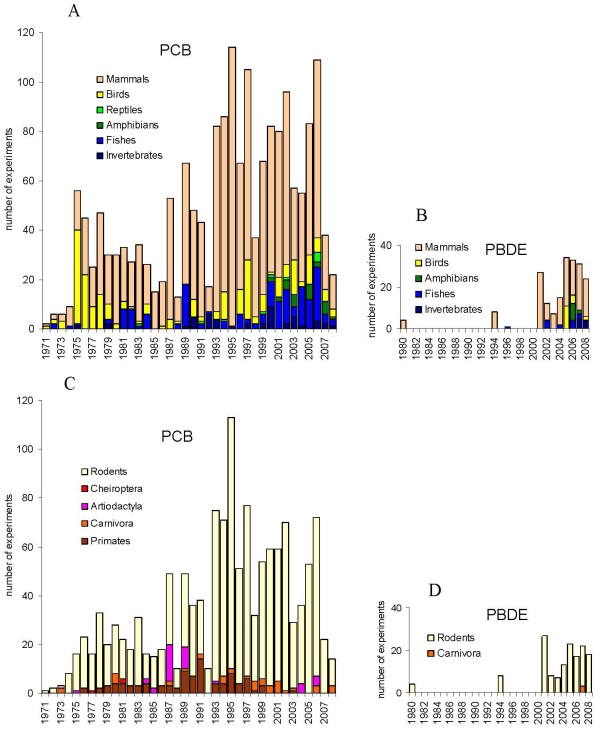
**Distribution of animal species in experiments addressing PCB (A, C) and PBDE (B, D) toxicity. **A, B – big taxonomic groups, C, D – mammalian models.

The diversity of the taxonomic groups used as animal models for both PCB and PBDE studies is shown in Figure 6, including detailed mammalian models. While there is some diversification of large taxonomic groups used as animal models over the last years, there is also an opposite trend within mammalian models.

Rodents are thus becoming the sole mammalian order used in most toxicological experiments. Each animal model presents advantages and limitations depending on the endpoints of interest. Advantages and role of rodent models in toxicological studies are well known. The use of the rodent models only in experimental toxicology is not sufficient however, since rodent models have some limitations, not covered in modern studies by any other species. For example, speaking about PCB and PBDE it has to be mentioned, that organohalogen toxicokinetics is very different in rodents and humans [82]. Rodents differ also in a number of important parameters for the study of developmental toxicity [83], including physiology of pregnancy, pre- and neonatal development.

Although primates are the ideal model to simulate human physiology, development and toxicokinetics, their use is dwindling for ethical and financial considerations. An important problem stems from the fact that no other group with human-like parameters is used in most toxicological studies. Although having limitation as precocial species certain mammals of the *Artiodactyla *order (even-toed ungulates) are presumably good models for simulating human-like pregnancy and toxicokinetics [84], there are unfortunately few reports of PCB studies and none, to our knowledge, of PBDE studies using hoofed mammals. The detailed discussion of animal species better corresponding to diverse demands of thorough simulation of humans in toxicological experiments is out of frames of this survey. The revealed tendency of using just mice and rat is obviously negative.

### Quality control: the internal dose in exposed animals?

The choice of dosing paradigm is a challenge. Estimation of the internal dose is thus a means of "quality control" and yields valuable information for the transfer of data from animal to human studies [85].

**Figure 7:**
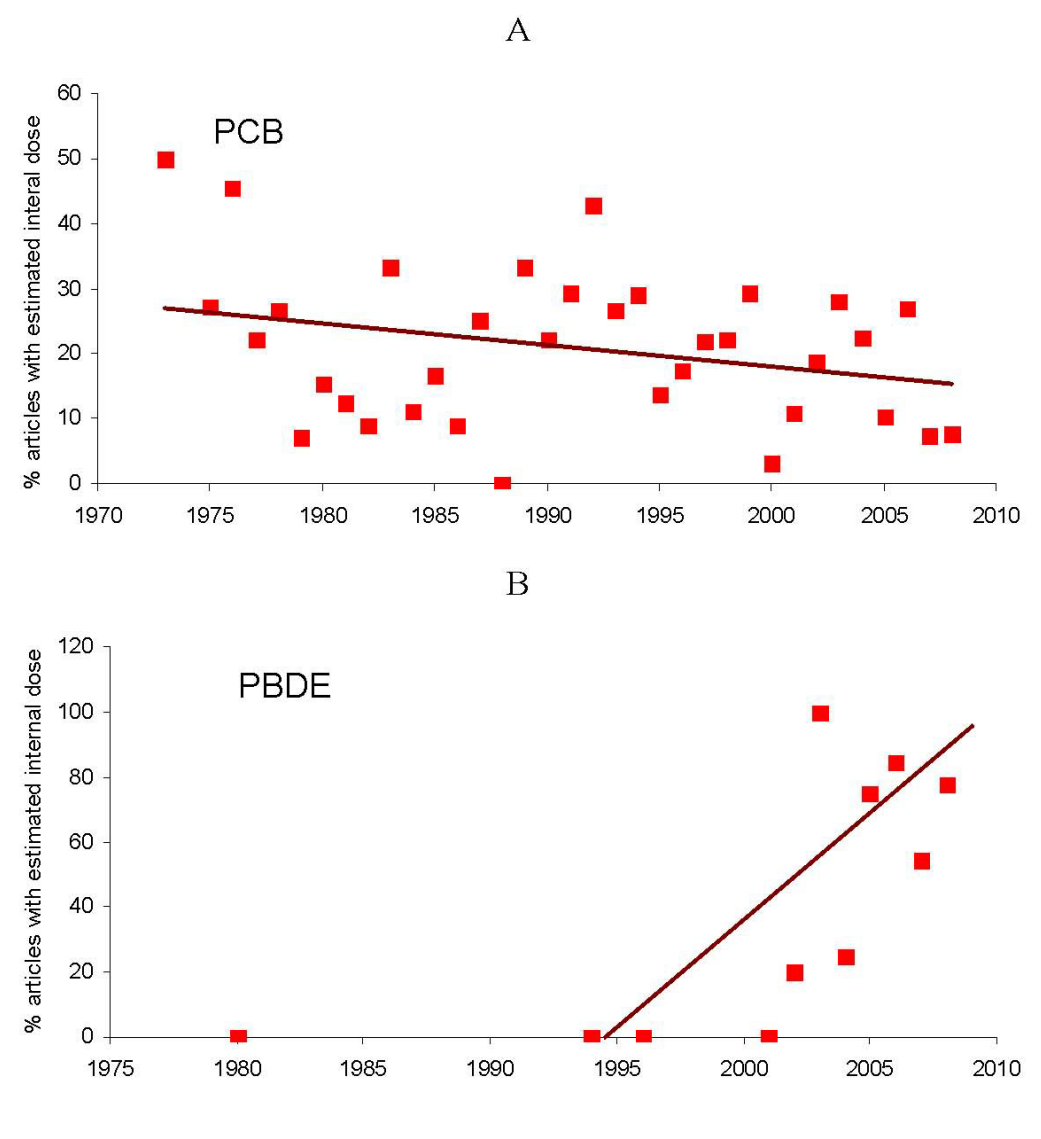
**Temporal trends in percent of studies using estimation of internal dose in animal models.** A – studies addressing PCB toxicity; B – studies addressing PBDE toxicity.

The data on estimation of internal doses in PBDE and PCB studies are summarized in Figure 7. This documents that lesson from PCB investigations was exceptionally transferred to PBDE studies. However, whereas measurement of internal dose is increasing rapidly in PBDE studies (*β *= 6.32; *p *= 0.01), it surprisingly tends to decrease slowly in PCB studies (*β *= -0.33; *p *= 0.09).

### Choosing the best route of exposure

The two major routes of exposure used in experiments with animals are the oral routes and *via *injections. Both routs named here are generic as oral administration may be performed by oral gavage or by addition of the substance to food; injections include subcutaneous, intravenous and intraperitoneal and may be further divided by point of injection. Each certain type of substance administration has its unique advantages and disadvantages. However the main advantage of all oral routes is that they better mimic natural conditions of exposure (at least for organohalogens) and are less stressful for animals. Oral routes add uncertainty to the estimation of exposure since rate of absorption and transformation of substance in the intestine are often poorly known. On the contrary generic routes *via *injections allow for better control of the internal dose, especially at low doses. They do not simulate in majority of cases environmental exposures however and are stressful.

**Figure 8:**
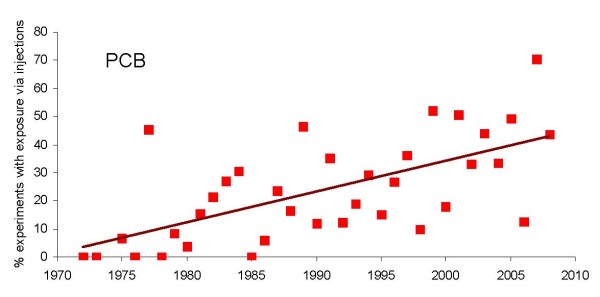
Temporal trend towards increase in number of exposures *via *injections *versus *oral exposures in PCB experiments.

According to the data summarized in Figure 8, there is a clear tendency to transfer from oral routes to injections in PCB studies (*β *= 1.09; *p *< 0.0001). Surprisingly, only 4 PBDE studies reported using injections. Thus PCB and PBDE studies seem to be at odds with respect to route of exposure.

## Conclusion

Critical analysis of reports of PCB and PBDE toxicity testing in animal models shows that the logistics of toxicological experiments makes animal data difficult to transfer to human studies. Moreover, analysis of the history of animal studies shows that the hindsight offered by PCB experimental history had little impact on experimental toxicology and future investigations into PBDE. Indeed the studies on PBDE display the same flaws as their PCB counterparts, indicating that lessons were not drawn from the history of PCB.

One important inconsistency between animal and human research was shown recently in a critical review of PCB experimental studies [85]: the lack of animal data on several persistent congeners which are currently used as a measure of human exposure while experimental studies in animals are frequently conducted with commercial PCB mixtures, a test design that does not reflect the exposure situation in humans.

For animal-model data to benefit human studies, our survey highlights that the following important lessons must be drawn.

• Intensive animal testing must precede marketing of a new substance or be concomitant to its arrival upon the market in order to avoid contamination of the environment and human population by chemicals with unknown hazardous properties. As research activity is dependent on funding this lesson is relevant not only for toxicologist but for organizations providing financial support.

• Toxic effects in experimental studies are obtained at levels of exposure several orders of magnitude higher than exposure levels of the general population, preventing direct transfer of animal results to human studies. Hence, there is a need for toxic-substances testing with doses relevant to human exposures and measurement of internal dose.

• There is a need to use animal species with human-like toxicokinetics and physiology of development and pregnancy. Rodents are becoming the sole model for toxicological experiments. Toxic effects investigation, especially in developmental toxicology, should extend to other mammalian species.

• For endocrine disruptors, all endocrine glands must be addressed. The role of environmental contaminants in increasingly prevalent endocrine disorders such as childhood obesity and diabetes mellitus is an important research avenue.

• According to the existing paradigm, investigation into the most sensitive stages of development occurs in the following sequence: adult animals – neonates – foetuses. However, the reverse sequence would accelerate data accumulation for risk assessment.

• Animal experiments specifically designed to uncover outcomes suitable for epidemiological research are needed. There is increasing demand for this information to design human studies.

We conclude that poor integration of the human and experimental branches of toxicology can and does hamper use of data obtained in experiments with animal models. Hence, broadening of the dialogue between the various branches of the field of toxicology is needed.

## Abbreviations

ATSDR: US Agency for Toxic Substances and Disease Registry; BDE-47: 2,2',4,4'-tetrabromodiphenyl ether; deca-BDE: deca-bromodiphenyl ether; NOAEL: no observed adverse effect level; octa-BDE: octa-bromodiphenyl ether; PBDE: polybrominated diphenyl ether; PBPK: physiologically-based pharmacokinetic; PCB: polychlorinated biphenyl; PND: postnatal day; PVC: polyvinyl chloride.

## Competing interests

The authors declare that they have no competing interests.

## Authors' contributions

AS has made substantial contributions to conception, design of review, acquisition, analysis and interpretation of data; LT has been involved in drafting the manuscript and revising it critically. Both authors have approved the final version of the manuscript.

## Supplementary Material

Additional file 1 **Includes data (authors, date publishing, used model organisms, doses, exposed life stages, endpoints, internal dose measurement, type of substance administration) on articles addressing health effects of PCB toxicity in experiments with animal models that were selected according to the criteria described in background.** Click here for file

Additional file 2 **Includes data (authors, date publishing, used model organisms, doses, exposed life stages, endpoints, internal dose measurement, type of substance administration) on articles addressing health effects of PBDE toxicity in experiments with animal models that were selected according to the criteria described in background.** Click here for file
